# Supplementary material for: Pesticide Residue Trends in Fruits and Vegetables from Farm to Fork in Kampala Metropolitan Area, Uganda—A Mixed Methods Study
Source: Int J Environ Res Public Health. 2022 Jan 26;19(3):1350. doi: 10.3390/ijerph19031350 (PMC8835516; doi:10.3390/ijerph19031350)
Supplement: Supplementary file 1 [file ijerph-19-01350-s001.zip › ijerph-1498057-supplementary (1).pdf]

Table S1: Pesticide active ingredients analysed in fruits and vegetables categorized by class, use and WHO classification.

Pesticide Active Ingredient	Class	Main Use	WHO classification
Dithiocarbamates	Diothiocarbamates	I	O
Omethoate	OP	I	Ib
Methamidophos	OP	I	Ib
Acephate	OP	I	II
Monocrotophos	OP	I	Ib
Vamidothion	OP	I	Ib
Dimethoate	OP	I	II
Mevinphos	OP	I	Ia
Phosphamidon	OP	I	Ia
Fonofos	OP	I	O
Azamethiphos	OP	I	II
Dichlorvos	OP	I	Ib
Malaoxon	OP	I	Ib
Methidathion	OP	I	Ib
Azinphosmethyl	OP	I	Ib
Malathion	OP	I	III
Methacrifos	OP	I	II
Propetamophos	OP	I	II
Triazophos	OP	I	Ib
Mecarbam	OP	I	Ib
Azinphosethyl	OP	I	Ib
Isofenphosmethyl	OP	I	Ib
Ethoprophos	OP	I	Ia
Fenamiphos	OP	N	Ib
Quinalphos	OP	I	II
Coumaphos	OP	AC	Ib
Chlorfenvinphos	OP	I	Ib
Chlorpyriphos-methyl	OP	I	II
Temephos	OP	I	III
Profenofos	OP	I	II
Terbufos	OP	I	Ia
Ethion	OP	I	II
Phosalone	OP	I	II
Pirimiphosmethyl	OP	I	II
Fenitrothion	OP	I	II
Chlorpyriphos	OP	I	II
Diazinon	OP	I	II

Aminocarb	C	I	II
Methomyl	C	I	Ib
Aldicarb	C	I	Ia
Butocarboxim	C	I	Ib
Aldicarbfragment	C	I	Ia
Pirimicarb	C	I	II
Dioxacarb	C	I	O
Carbaryl	C	I	II
Carbofuran	C	I	Ib
Thiodicarb	C	I	II
Methiocarb Mercaptodimethur	C	I	Ib
Alanycarb	C	I	II
Benfuracarb	C	I	II
Carbosulfan	C	I	II
Methiocarb	C	I	Ib
Imidacloprid	N	I	II
Acetamiprid	N	I	II
Thiacloprid	N	I	II
Bifenthrin	PY	I	II
LambdaCyhalothrin	PY	I	II
Deltamethrin	PY	I	II
Cypermethrin	PY	I	II
Ethirimol	O (Pyrimidinol)	F	U
Glyphosate	O (Phosphonoglycine)	H	III
Carbendazim	O (Benzimidazole)	F	U
Carboxin	O (Oxathiin)	F	III
Imazalil	O (Imidazole	F	II
Diuron	O (Phenylamide)	H	III
Metazachlor	O (Chloroacetamide)	H	III
Metalaxyl	O (Phenylamide)	F	III
Azaconazole	O (Triazole)	F	II
Dimethachlor	O (Chloroacetamide)	H	II
Clomazone	O (Isoxazolidinone)	H	II
Azoxystrobin	O (Strobilurin)	F	U
Pyrimethanil	O (Anilinopyrimidine	F	U
Fludioxonil	O (Phenylpyrrole)	F	U
BosclidNicobifen	O (Carboxamide)	F	U
Triadimenol	O (Triazole)	F	II
Spirotetramat	O (Tetramic acid)	I	III
Fenhexamid	O (Hydroxyanilide)	F	U

Fenarimol	O (Pyrimidine)	F	III
Fipronil	O (Phenylpyrazole)	I	II
Bupirimate	O (Pyrimidinol)	F	III
Flubendiamide	O (Benzene-dicarboxamide)	I	O
Metolachlor	O (Chloroacetamide)	H	III
Cyprodinil	O (Anilinopyrimidine)	F	O
Benalaxyl	O (Acylamino acid)	F	III
Prochloraz	O (Imidazole	F	II
Metconazole	O (Triazole)	F	II
Fluazifop	O (Aryloxyphenoxypropionate)	H	O
Diniconazole	O (Triazole)	F	II
Flufenoxuron	O (Benzoylurea)	I	III
Pyriproxyfen	O (Unclassified)	I	U
Quinoxyfen	O (Quinoline)	F	U
Proquinazid	O (Quinazolinone)	F	U
Fenazaquin	O (Quinazoline)	AC	II

OP – Organophosphate, C – Carbamate, N – Neonicotinoids, PY – Pyrethroids, O – Others, I – Insecticide, F – Fungicide, H – Herbicide, AC – Acaricide, O – Obsolete, Ia – Extremely hazardous, Ib – Highly hazardous, II – Moderately hazardous, III – Slightly hazardous, U – Unlikely to present acute hazards

Figures S1–S21: Graphs showing terend of pesticide residue concentrations (µg/Kg) along the supply (farm to fork) chain (1 – Farm, 2 – Market, 3 – Street, 4 – Restaurant and 5 – Home)

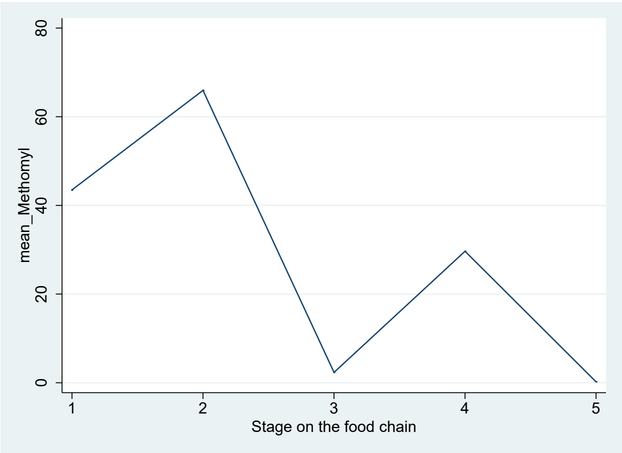

Figure S1: Trend of Methomyl along the chain

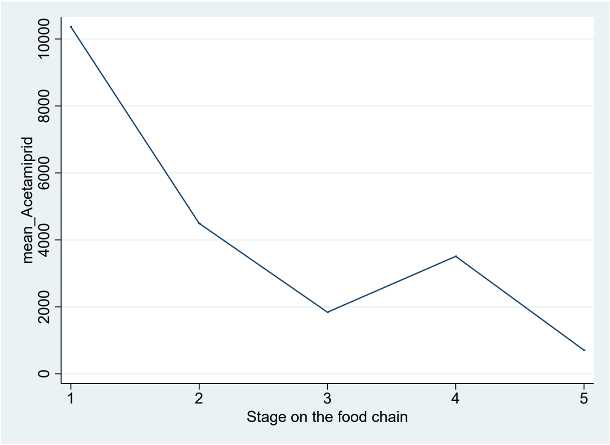

Figure S2: Trend of acetamiprid along the chain

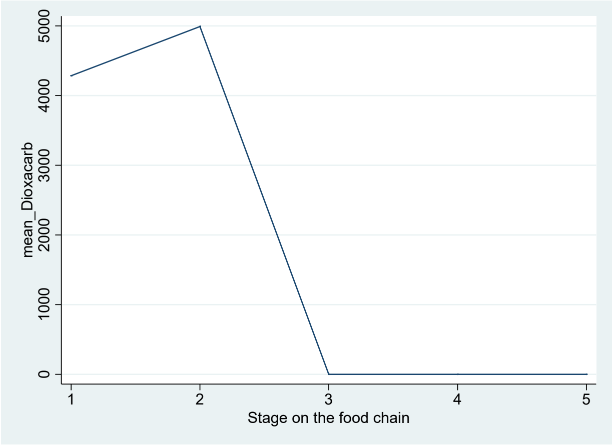

Figure S3: Trend of dioxacarb along the chain

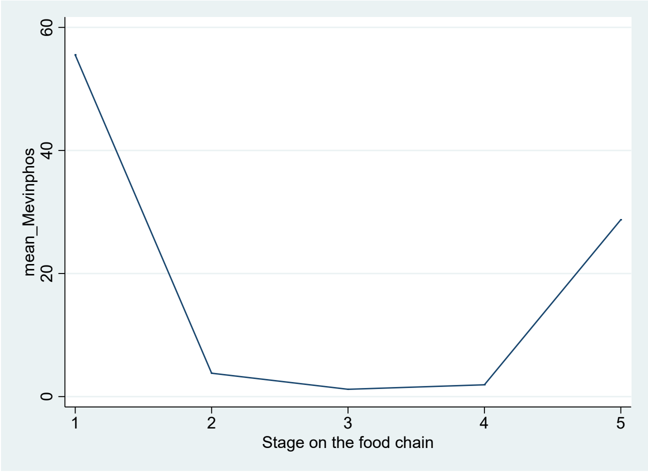

Figure S4: Trend of mevinphos along the chain

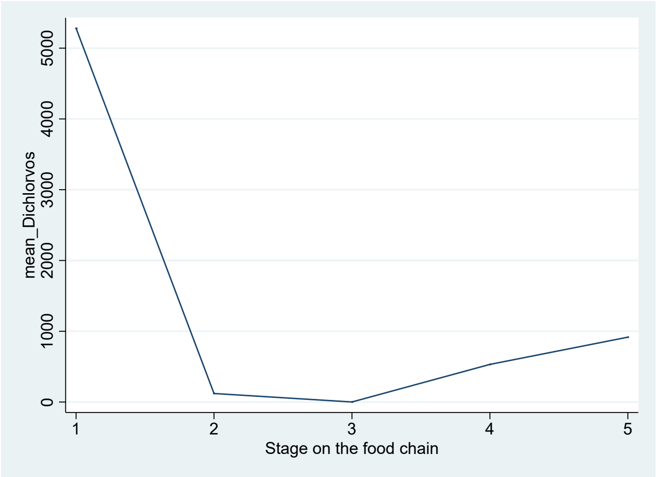

Figure S5: Trend of dichlorvos along the chain

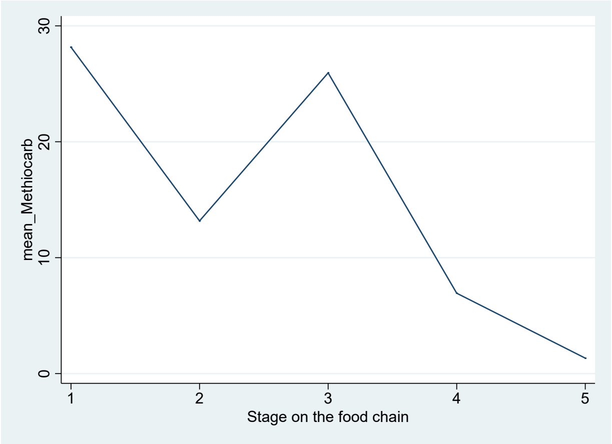

Figure S6: Trend of methiocarb along the chain

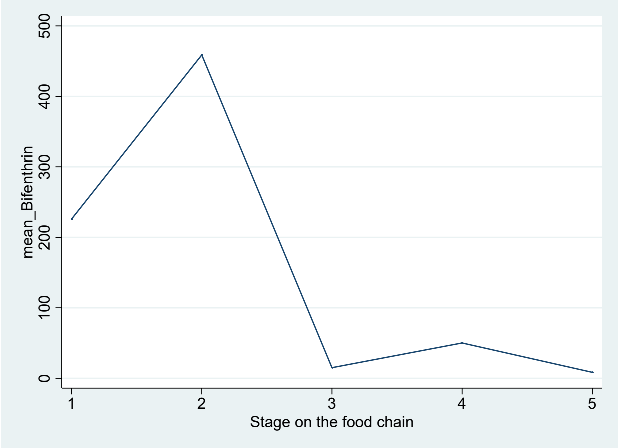

Figure S7: Trend of bifenthrin along the chain

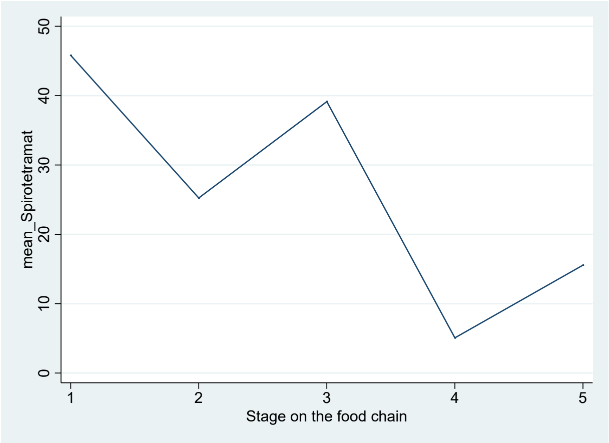

Figure S8: Trend of spirotetramat along the chain

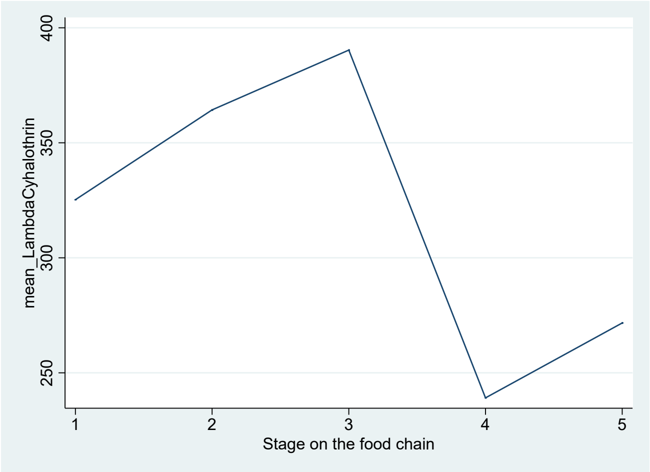

Figure S9: Trend of lambda-cyhalothrin along the chain

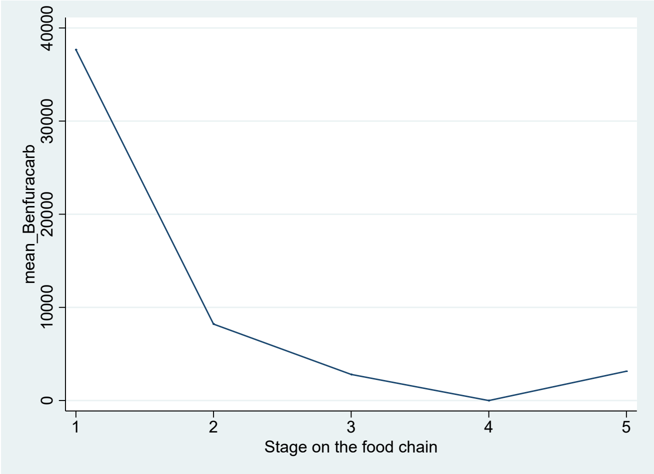

Figure S10: Trend of Benfuracarb along the chain

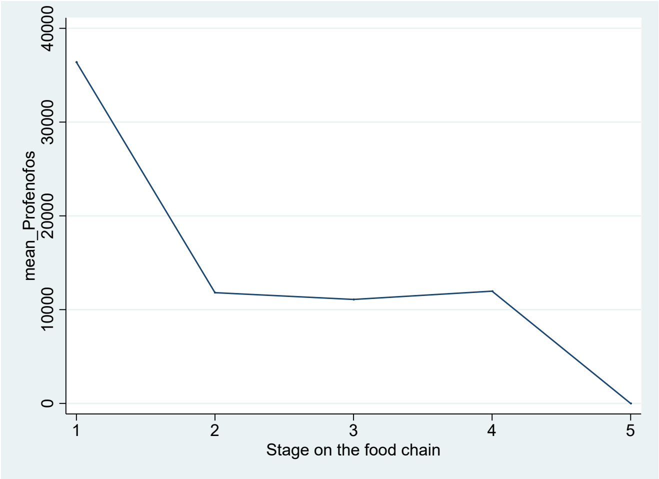

Figure S11: Trend of profenofos along the chain

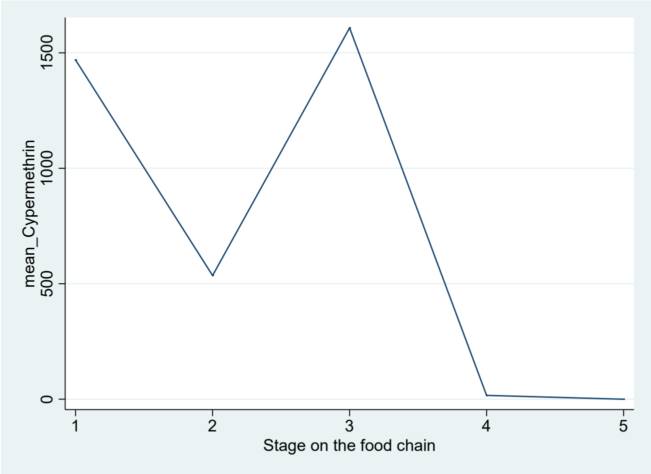

Figure S12: Trend of cypermethrin along the chain

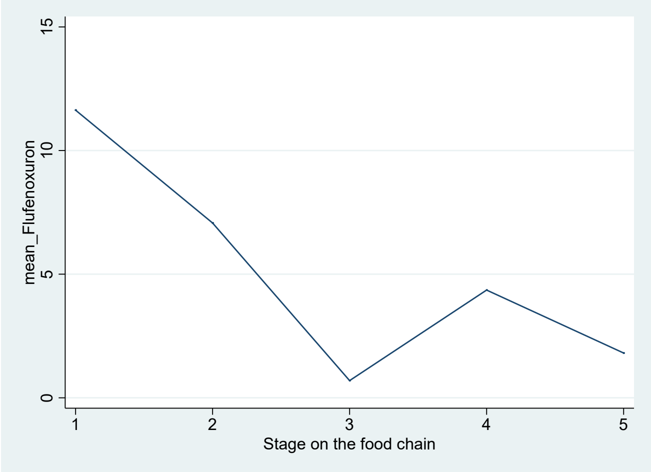

Figure S13: Trend of flufenoxuron along the chain

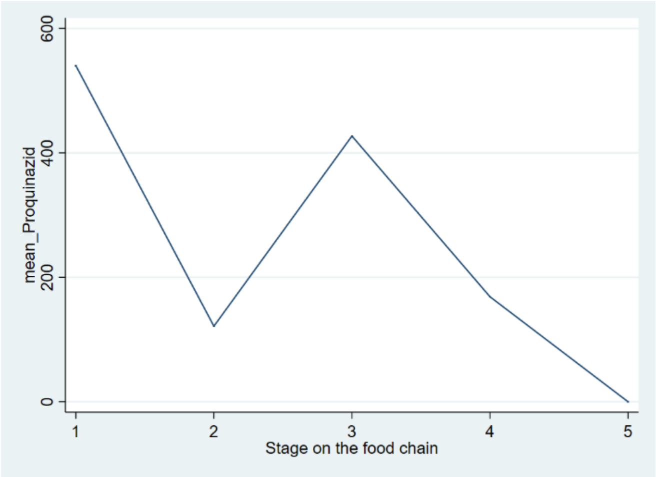

Figure S14: Trend of proquinazid along the chain

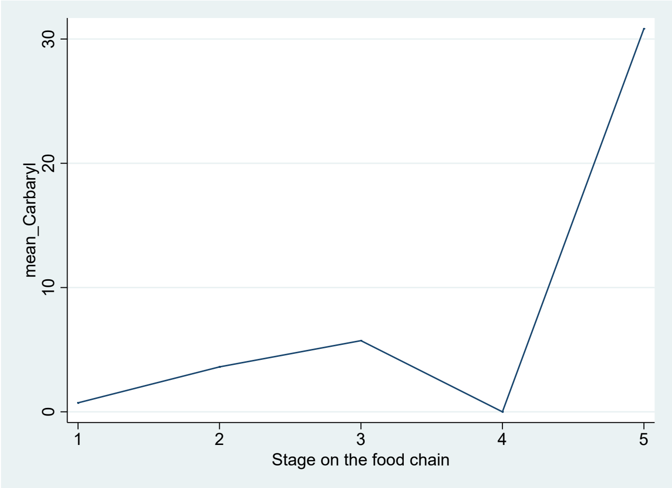

Figure S15: Trend of carbaryl along the chain

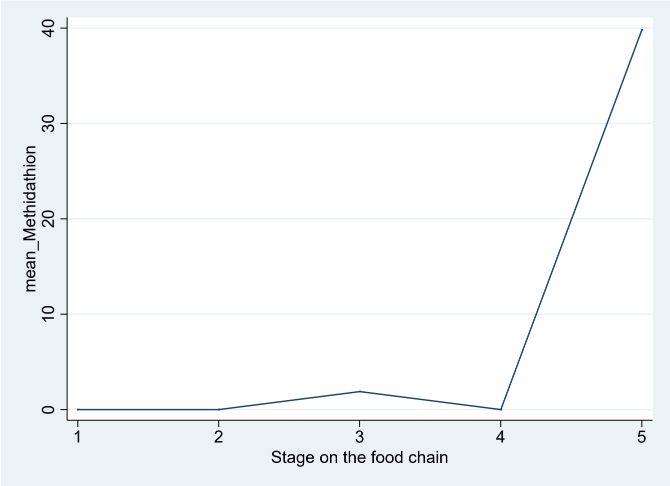

Figure S16: Trend of methidathion along the chain

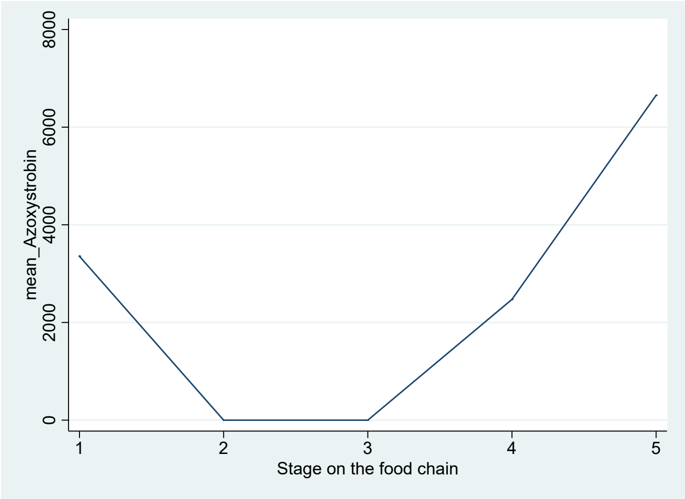

Figure S17: Trend of azoxystrobin along the chain

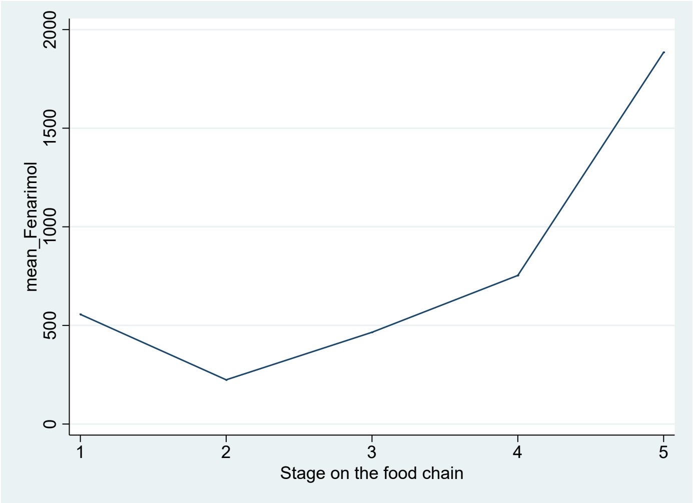

Figure S18: Trend of fenarimol along the chain

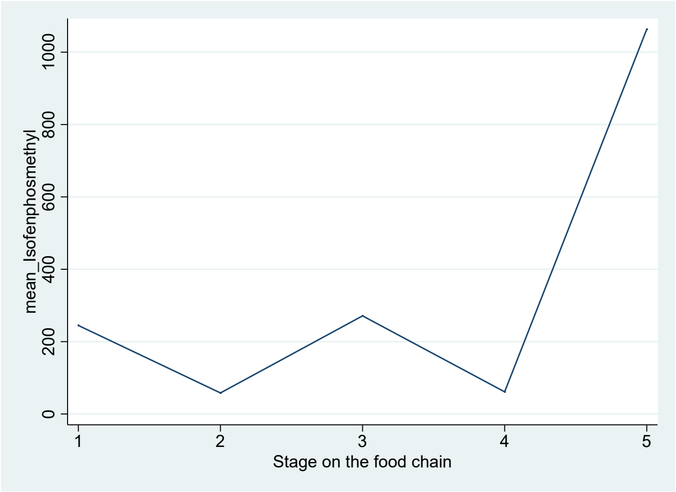

Figure S19: Trend of isofenphosmethyl along the chain

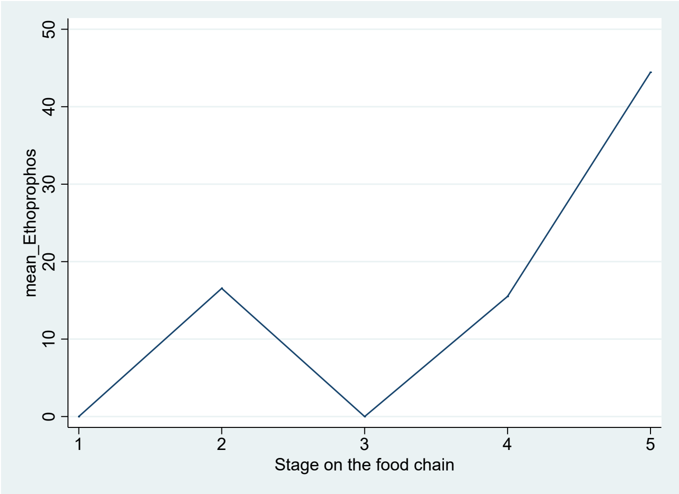

Figure S20: Trend of ethoprophos along the chain

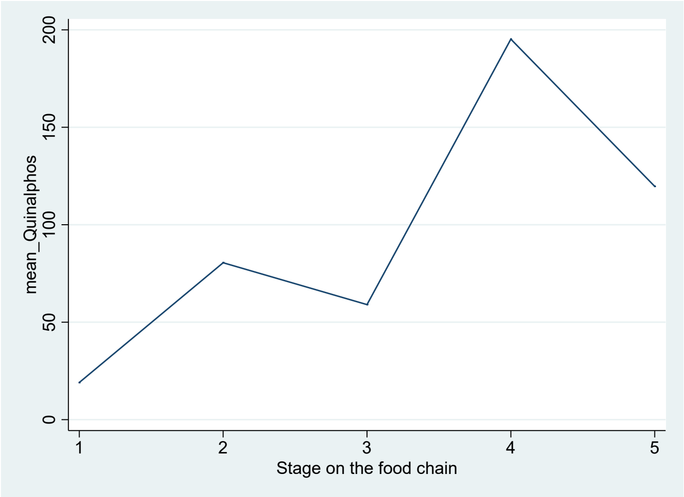

Figure S21: Trend of quinalphos along the chain

Table S2: Pesticide residue concentration in fruit and vegetable samples by stage of sampling along the supply chain.

Pesticide		Farm			Market			Street			Restaurant			Home		
	LOD (ng/kg)	DF	p95	Max	DF	p95	Max	DF	p95	Max	DF	p95	Max	DF	p95	Max
Dithiocarbamate[a]	5.6	22	2462.6	3153.1	29	1631.8	2829.6	11	2966.7	3597.7	7	2800.8	3920.9	8	1282.7	1376.1
**Organophosphate**																
Omethoate	14.6	2	-	321.9	3	1891.8	12420.3	ND	-	-	5	3712.3	5970.6	2	48.6	97.1
Acephate	26.1	17	5847.8	8441.3	19	2889.8	17761.3	3	699.7	735.7	7	1262.8	17.45.9	6	653.6	80-
Monocrotophos	12.4	13	129.3	173.0	8	239.0	501.2	3	269.6	434.7	6	261.1	278.4	5	135.3	185.1
Vamidothion	10.0	6	80.9	717.4	13	295.3	345.2	5	10.5	20.9	4	46.3	62.7	2	50.4	100.7
Dimethoate	8.0	ND	-	-	1	-	23197.5	ND	-	-	ND	-	-	ND	-	-
Mevinphos	33.9	9	307.6	640.2	6	95.1	771.5	2	37.6	63.4	3	34.1	54.4	1	143.7	287.4
Phosphamidon	16.6	2	-	65.0	5	37.8	319.0	2	154.8	296.1	ND	-	-	2	34.2	52.7
Fonofos	14.2	17	843221.2	1013911	17	993663.9	2590684	4	6695.6	6892.0	4	1092929	1111318	4	688902.7	922173.1
Azamethiphos	5.4	4	48.5	100.1	2	-	510.1	3	21.2	36.3	3	73.2	86.9	ND	-	-
Dichlorvos	15.3	11	57879.7	115247.9	6	913.2	1917.2	4	439.2	575.2	4	2399.9	4308.9	5	3521.0	4043.3
Malaoxon	14.6	3	12.5	231.8	6	36.7	201.1	1	539.1	1078.2	2	111.5	179.7	3	112.2	147.6
Methidathion	14.4	ND	-	-	ND	-	-	1	18.9	18.9	ND	-	-	2	353.0	353.0
Malathion	19.5	2	-	2336.1	ND	-	-	ND	-	-	ND	-	-	1	49.3	98.5
Methacrifos	5.3	ND	-	-	2	-	1049.2	ND	-	-	ND	-	-	ND	-	-
Propetamophos	8.1	23	33590.7	33691.9	13	37180.7	43208.9	1	36295.5	36295.5	4	25885.6	25885.6	2	23638.2	23638.2
Isofenphosmethyl	20.0	5	1853.1	2152.2	3	347.5	1057.0	1	2712.9	2712.9	1	613.0	613.0	4	4616.0	4616.0
Ethoprophos	84.6	ND	-	-	1	-	413.6	ND	-	-	1	155.1	155.1	1	444.4	444.4

Fenamiphos	9.4	1	-	3.2	3	8.2	31.8	4	54.5	54.5	2	26.9	26.9	ND	-	-
Quinalphos	31.8	2	177.6	298.8	5	503.1	759.9	1	590.3	590.3	3	1550.3	1550.3	5	431.6	431.6
Coumaphos	15.8	1	-	7.2	ND	-	-	ND	-	-	ND	-	-	ND	-	-
Chlorpyriphos-methyl	7.8	4	342.6	2743.9	ND	-	-	1	47.0	93.9	3	1032.7	1488.8	ND	-	-
Temephos	7.6	ND	-	-	1	-	138.7	ND	-	-	2	53.2	53.2	1	208.6	208.6
Profenofos	9.7	5	159129.2	406420.3	8	20856.4	261739.8	3	55452.2	85699.6	4	86547.0	119716.8	2	7222.0	11199.8
Pirimiphosmethyl	19.4	ND	-	-	1	-	206.6	ND	-	-	ND	-	-	ND	-	-
Fenitrothion	10.4	9	65045.6	495132.2	6	79269.0	180716.1	5	326975.6	505552.9	6	228621.4	289100.4	2	43378.4	63061.5
**Carbamates**																
Aminocarb	17.9	10	91.1	1734.3	10	95.3	22398.6	4	133.4	211.0	4	109.5	110.4	5	84.8	111.6
Methomyl	33.5	11	273.7	354.1	15	343.3	532.9	2	71.4	119.4	4	309.0	428.7	2	46.1	90.1
Aldicarbfragment	13.0	5	279.5	419.3	7	167.1	189.7	2	256.6	481.0	2	147.1	266.1	2	162.5	272.7
Pirimicarb	26.0	14	191.1	441.2	8	33.1	578.4	ND	-	-	5	539.7	690.3	4	80.9	84.8
Dioxacarb	13.1	7	39913.3	104497.4	7	45256.5	79514.3	1	34623.7	69247.3	ND	-	-	ND	-	-
Carbaryl	7.7	3	6.8	195.1	3	48.5	90.4	1	28.6	57.3	ND	-	-	2	154.1	201.9
Carbofuran	8.7	3	61.8	149.0	1	-	9.4	1	480.9	961.8	2	396.0	784.9	1	4.7	9.4
Alanycarb	13.1	6	100872.2	153428	8	102749.4	209392.6	3	68856.3	68656.3	3	114102.6	114102.6	6	146164.8	146164.8
Benfuracarb	50.0	4	48653.6	878508.6	3	52691.9	142734.9	3	15620.5	15620.5	1	8.1	8.1	1	31466.9	31466.4
Methiocarb	43.9	7	205.2	320.9	4	74.4	172.6	4	149.6	149.6	2	60.2	60.2	2	9.5	9.5
**Neonicotinoids**																
Imidacloprid	34.0	10	2962.4	5324.8	19	1693.9	4951.2	8	5044.8	8118.0	8	6404.2	7547.0	5	1061.7	1146.7
Acetamiprid	20.4	6	47208.4	126623.4	13	21362.3	58245.6	5	13271.6	18400.2	4	17544.0	18985.4	2	8106.3	9162.5
Thiacloprid	10.5	3	28.0	192	ND	-	-	ND	-	-	ND	-	-	ND	-	-

**Pyrethroid**																
Bifenthrin	18.7	11	803.0	2529.2	13	1439.3	6843.6	1	75.2	150.3	4	216.5	263.2	2	42.3	46.5
Lambda-Cyhalothrin	21.1	19	879.3	1840.9	19	878.2	2478.6	7	951.3	989.3	10	741.6	895.1	9	701.1	935.7
Deltamethrin	10.0	2	-	164.6	1	-	1786.7	ND	-	-	ND	-	-	ND	-	-
Cypermethrin	11.1	8	8981.3	15614.1	12	1302.6	7553.2	5	7983.7	12092.1	5	1546.8	1983.0	3	979.5	1519.8
**Others**																
Carbendazim	15.1	2	-	69.5	4	128.8	4155.4	4	25.7	25.8	4	91.0	99.0	2	8.1	16.2
Imazalil	13.8	12	2106.2	3911.5	12	1316.1	1724.5	4	4896.6	5862.7	2	3970.7	7639.5	4	2619.9	3042.3
Metazachlor	14.6	11	122.3	797.9	12	130.9	1018.0	7	204.5	209.5	5	196.6	269.6	5	117.3	134.0
Metalaxyl	22.5	1	-	119.2	1	-	1442.0	ND	-	-	ND	-	-	ND	-	-
Azaconazole	5.5	2	14.1	197.5	6	401.9	1594.4	4	345.2	345.2	ND	-	-	2	434.0	434.0
Clomazone	7.4	1	-	50.7	ND	-	-	ND	-	-	ND	-	-	ND	-	-
Azoxystrobin	7.4	2	32983.7	50917.8	ND	-	-	ND	-	-	2	18365.7	18365.7	1	66525.7	66525.7
Pyrimethanil	17.7	7	173.1	550.9	13	384.7	395.3	5	583.9	583.9	5	292.4	292.4	8	731.5	731.5
Spirotetramat	18.4	9	67.0	963.9	3	115.1	477.8	2	286.0	286.0	2	32.5	32.5	4	71.3	71.3
Fenhexamid	14.6	3	144773.6	168164.4	3	205560	369295.7	2	493661.6	493661.6	1	14952.0	14952.0	4	64185.3	64185.3
Fenarimol	13.1	12	2045.9	5985.5	9	419.8	4148.9	5	2048.9	2649.1	5	3424.7	3429.2	4	7056.4	10385.2
Fluazifop	16.8	2	-	3612.9	2	-	171043.9	ND	-	-	ND	-	-	ND	-	-
Flufenoxuron	15.4	6	41.2	178.1	6	19.7	78.1	1	3.5	7.0	3	20.6	34.9	1	9.1	18.1
Pyriproxyfen	6.6	ND	-	-	ND	-	-	1	50.2	50.2	ND	-	-	ND	-	-
Quinoxyfen	27.5	5	198.5	534.3	1	-	254.3	1	463.2	463.2	2	17.5	17.5	1	469.2	469.2
Proquinazid	14.6	3	4021.2	7990.3	1	-	3033.8	1	4270.3	4270.3	1	1687.1	1687.1	ND	-	-

a – Mancozeb, Maneb, Metiran, Pronineb, Thiram and Zinam expressed in CS_2, LOD – Limit of Detection, DF – Detection Frequency, ND – Not Detected, p95 – 95th Percentile, Max – Maximum concentration
